# Supplementary material for: FOXO1-mediated lipid metabolism maintains mammalian embryos in dormancy
Source: Nat Cell Biol. 2024 Jan 4;26(2):181–93. doi: 10.1038/s41556-023-01325-3 (PMC10866708; doi:10.1038/s41556-023-01325-3)
Supplement: Supplementary file 1 — Supplementary table legends. [file 41556_2023_1325_MOESM1_ESM.pdf]

nature cell biology

Article

https://doi.org/10.1038/s41556-023-01325-3

FOXO1-mediated lipid metabolism maintains mammalian embryos in dormancy

In the format provided by the authors and unedited

This PDF file includes the legends for Supplementary Tables 1 to 11.

Supplementary Table 1

Proteomics dataset of ESCs and TSCs into pausing and release from pausing. MaxQuant software (v1.6.10.43).

Supplementary Table 2

DEP output for entry into pausing compared to normal and divergence scores for all 327 KEGG pathways for ESCs and TSCs. Differentially expressed genes were identified using the DEP package (p-value of <0.05).

Supplementary Table 3

GO output for entry into pausing of ESCs (immediate and adaptive changes for up and down-regulated proteins) and TSCs. The Benjamini-Hochberg correction was used. p-value cutoff: 0.05, q-value cutoff: 0.1.

Supplementary Table 4

ESCs bulk metabolomics (both cell number and protein concentration normalized). MultiQuantTM software v.2.1.1 was used and metabolites with a p-value <0.05 and absolute log_2FC >0.75 was regarded as statistically significant.

Supplementary Table 5

Media compositions

Supplementary Table 6

Complete proteomics results of single embryos. MaxQuant software (v1.6.10.43) was used.

Supplementary Table 7

DEP package output for single embryo data at D5 of pausing (p-value of <0.05).

Supplementary Table 8

KEGG pathway expression analysis at D5 of pausing.

Supplementary Table 9

Significantly up- or down-regulated genes in at least 4 out of 6 dormant adult cells. DESeq was used to call DE genes with a cutoff of padj < 0.05.

Supplementary Table 10

GO output for dormant versus proliferating stem cells. The Benjamini-Hochberg correction was used. p-value cutoff: 0.05, q-value cutoff: 0.1.

Supplementary Table 11

Exact p values derived from statistical tests
