# Supplementary material for: FOXO1-mediated lipid metabolism maintains mammalian embryos in dormancy
Source: Nat Cell Biol. 2024 Jan 4;26(2):181–93. doi: 10.1038/s41556-023-01325-3 (PMC10866708; doi:10.1038/s41556-023-01325-3)
Supplement: Supplementary file 2 — Reporting Summary [file 41556_2023_1325_MOESM2_ESM.pdf]

nature portfolio

Corresponding author(s): Aydan Bulut-Karslioglu

Last updated by author(s): Nov 14, 2023

Reporting Summary

Nature Portfolio wishes to improve the reproducibility of the work that we publish. This form provides structure for consistency and transparency in reporting. For further information on Nature Portfolio policies, see our Editorial Policies and the Editorial Policy Checklist.

Statistics

For all statistical analyses, confirm that the following items are present in the figure legend, table legend, main text, or Methods section.

n/a	Confirmed	
☐	☒	The exact sample size (*n*) for each experimental group/condition, given as a discrete number and unit of measurement
☐	☒	A statement on whether measurements were taken from distinct samples or whether the same sample was measured repeatedly
☐	☒	The statistical test(s) used AND whether they are one- or two-sided<br>*Only common tests should be described solely by name; describe more complex techniques in the Methods section.*
☒	☐	A description of all covariates tested
☐	☒	A description of any assumptions or corrections, such as tests of normality and adjustment for multiple comparisons
☐	☒	A full description of the statistical parameters including central tendency (e.g. means) or other basic estimates (e.g. regression coefficient) AND variation (e.g. standard deviation) or associated estimates of uncertainty (e.g. confidence intervals)
☐	☒	For null hypothesis testing, the test statistic (e.g. *F*, *t*, *r*) with confidence intervals, effect sizes, degrees of freedom and *P* value noted<br>*Give P values as exact values whenever suitable.*
☒	☐	For Bayesian analysis, information on the choice of priors and Markov chain Monte Carlo settings
☒	☐	For hierarchical and complex designs, identification of the appropriate level for tests and full reporting of outcomes
☐	☒	Estimates of effect sizes (e.g. Cohen's *d*, Pearson's *r*), indicating how they were calculated

Our web collection on statistics for biologists contains articles on many of the points above.

Software and code

Policy information about availability of computer code

Data collection

For ESC and TSC proteomics, LC-MS/MS was carried out by nanoflow reverse phase liquid chromatography (Dionex Ultimate 3000, Thermo Scientific) coupled online to a Q-Exactive HF Orbitrap mass spectrometer (Thermo Scientific).
ESC metabolites were quantified by metabolite extraction and tandem LC-MS/MS measurements.
For embryo metabolite detection, Mass Spectrometry Imaging was performed with the AP-SMALDI5-Orbitrap MS.
For embryo proteomics, digests were loaded onto Evotip Pure (Evosep, Odense, Denmark), peptide separation was carried out by nanoflow reverse phase liquid chromatography (Evosep One, Evosep) using the Aurora Elite column (15 cm x 75 µm ID, C18 1.7 µm beads, IonOpticks, Victoria, Australia). The LC system was coupled to the timsTOF SCP mass spectrometer (Bruker Daltonics, Bremen, Germany).
The Agilent Seahorse XFp Analyzer was used for ESC and TSC energetics and the Mito Fuel Flex test.
The Tecnai Spirit transmission electron microscope (FEI) operated at 120 kV, equipped with a 4kx4k F416 CMOS camera (TVIPS) was used for ultra-structure analysis of the embryos.
The Zeiss Plan-Apochromat 20x/0.8 objective on the Zeiss LSM880 Airy microscope using Airy scan was used for lipid droplet quantifications, H4K16ac, OCT4, CDX2, FOXO1, c-CASPASE3, LaminB1, H3K9me2, KI67, p-mTOR, pS6, pAKT, pAMPK, pACC, pULK, LC3B, LAMP1, SOX2, and CPT1A. The Zeiss Plan-Apochromat 63x 1.4NA oil objective was used on the Zeiss LSM880 Airy microscope using Airy scan for LaminB1 and H3K9me2 imaging. Airy scan mode and image processing was done using Zen black software (version 2.3).

Data analysis

MaxQuant software (v1.6.10.43): Raw MS data processing.
Dia-NN (v1.8.1): Peptide search for embryo proteomics.
Data analysis and visualization: R (version 4.1.0), RStudio (version 1.3.1093 with R version 3.6.3)
survminer (version 0.4.9) and survival (version 3.3-1) packages for survival curves
Mass spec: Perseus (version 1.6.14.0), Dia-NN (v1.8.1), and DEP packages (version 1.14.0)
Destiny package (version 3.8.0): diffusion maps and pseudotime calculation.

MetaboAnalyst 4.0: time series analysis of proteomics data.
The R stats package “stats” (version 4.1.0): k-means clustering.
clusterProfiler (version 4.0.5): identification of enriched Biological Processes.
Plotting: ggplot2 (version 3.3.5) and R package ‘ComplexHeatmap’
MultiQuantTM software (version 2.1.1): relative quantification metabolomics data.
pheatmap package (version 1.0.12): heatmaps
Thermo imagequest software (version 1.1): Thermo RAW data analysis.
https://metaspace2020.eu: annotation and interpretation of MALDI-imaging data.
ImageJ (version 1.53): metabolite quantification
Wave software (version 2.4): Seahorse Mitostress and Glyostress programs.
ZEN Blue software (version 3.4): electron microscopy image analysis.
Fiji ImageJ2 (version 2.3.0): confocal image processing.
CellProfiler (version 4.2.1): confocal image quantifications
STAR-2.5.3a and HTSeq 0.11.4: RNAseq read alignmnent and count
DESeq2: differential gene expression

For manuscripts utilizing custom algorithms or software that are central to the research but not yet described in published literature, software must be made available to editors and reviewers. We strongly encourage code deposition in a community repository (e.g. GitHub). See the Nature Portfolio guidelines for submitting code & software for further information.

Data

Policy information about availability of data

All manuscripts must include a data availability statement. This statement should provide the following information, where applicable:

- Accession codes, unique identifiers, or web links for publicly available datasets
- A description of any restrictions on data availability
- For clinical datasets or third party data, please ensure that the statement adheres to our policy

UniProtKB and the mouse reference genome mm10 were used for peptide and read mapping. The proteomics datasets generated in this study have been deposited to the PRIDE database and are available via ProteomeXchange with the identifiers PXD033750, PXD033798, PXD041325. The metabolomics dataset has been deposited to peptideAtlas under the accession number PASS01758. Publicly available datasets used in this study are available under the accession numbers GSE126338, PRJEB13002, GSE121589, GSE101576, GSE116997, GSE138243, GSE138884, GSE135705, and GSE168617.

Human research participants

Policy information about studies involving human research participants and Sex and Gender in Research.

Reporting on sex and gender	N/A
Population characteristics	N/A
Recruitment	N/A
Ethics oversight	N/A

Note that full information on the approval of the study protocol must also be provided in the manuscript.

Field-specific reporting

Please select the one below that is the best fit for your research. If you are not sure, read the appropriate sections before making your selection.

☒ Life sciences ☐ Behavioural & social sciences ☐ Ecological, evolutionary & environmental sciences

For a reference copy of the document with all sections, see nature.com/documents/nr-reporting-summary-flat.pdf

Life sciences study design

All studies must disclose on these points even when the disclosure is negative.

Sample size	No statistical method was used to predetermine sample size, but our sample sizes are similar to those reported in previous publications13,76.<br>The number of replicates for each analysis is indicated in the text or figure legends.<br>For the ESC and TSC proteomics and ESC metabolomics experiment, three biological replicates were used.<br>For the embryo proteomics experiment, four to five biological replicates were used.<br>At least 5 embryos per condition were used for the MALDI-imaging experiments.<br>Four embryos per condition were used for electron microscopy.<br>For all confocal fluorescence imaging, at least 3 embryos were analyzed per condition. The number of analyzed embryos or cells is indicated in the figures.
Data exclusions	No data were excluded from the analysis.
Replication	The ESC and TSC proteomics and TSC metabolomics were performed with three biological replicates.<br>The embryo proteomics experiment was performed with four to five biological replicates.<br>The embryo supplementation experiments were performed once for supplements that did not improve developmental pausing length, and five times for carnitine supplementation experiments. The embryo staining experiments were performed with at least 3 biological replicates depending on the staining conditions. All attempts at replication were successful.
Randomization	For cell and embryo culture experiments, treatment groups were randomly attributed.
Blinding	No blinding was done because either the phenotype was obvious or treatment material needed to be refreshed often.

Reporting for specific materials, systems and methods

We require information from authors about some types of materials, experimental systems and methods used in many studies. Here, indicate whether each material, system or method listed is relevant to your study. If you are not sure if a list item applies to your research, read the appropriate section before selecting a response.

Materials & experimental systems

n/a	Involved in the study
☐	☒ Antibodies
☐	☒ Eukaryotic cell lines
☒	☐ Palaeontology and archaeology
☐	☒ Animals and other organisms
☒	☐ Clinical data
☒	☐ Dual use research of concern

Methods

n/a	Involved in the study
☒	☐ ChIP-seq
☒	☐ Flow cytometry
☒	☐ MRI-based neuroimaging

Antibodies

Antibodies used	Primary antibodies for stainings (dilutions for embryos/ESCs and TSCs):<br>Oct3/4 mouse anti-mouse antibody (Santa Cruz, cat.# sc-5279): 1:50/1:100<br>H4K16ac rabbit anti-mouse antibody (Millipore, cat.# 7329): 1:100<br>c-Caspase3 rabbit anti-mouse antibody (Cell Signaling Technology, cat.# 9661S): 1:100<br>FoxO1 rabbit anti-mouse antibody (Cell Signaling Technology, cat.# 2880T): 1:50<br>Anti-Lamin B1 antibody - Nuclear Envelope Marker (Abcam, cat#. 16048): 1:100/1:400<br>Anti-Histone H3 (di methyl K9) antibody (Abcam, cat.# 1220): 1:100/1:200<br>Ki67 mouse anti-mouse antibody (BD Pharmingen, cat.# 556003): 1:100<br>pmTOR rabbit anti-human antibody (Abcam, cat.# ab131538): 1:100/1:200<br>pS6 rabbit anti-mouse antibody (Cell Signaling Technology, cat.# 4858S): 1:100/1:200<br>pAKT rabbit anti-mouse antibody (Cell Signaling Technology, cat.# 4060T): 1:100/1:200<br>pAMPK rabbit anti-human antibody (Abcam, cat.# ab23875): 1:100<br>pACC rabbit anti-mouse antibody: (Cell Signaling Technology, cat.# 11818T): 1:100<br>pULK rabbit anti-mouse antibody: (Cell Signaling Technology, cat.# 14202S): 1:100<br>LC3B mouse anti-mouse antibody: (Cell Signaling Technology, cat.# 83506S): 1:100<br>LAMP1 rabbit anti-mouse antibody (Cell Signaling Technology, cat.# 99437S): 1:100<br>CPT1A mouse anti-mouse antibody (Abcam, cat.# ab128568): 1:100<br>SOX2 goat anti-mouse antibody (R&D System, cat.# AF2018): 1:100<br>CDX2 mouse anti-mouse antibody (Biogenex, cat.# MU392A-UC): -/1:200<br><br>Secondary antibodies (dilution for embryos/ESCs and TSCs):<br>donkey anti-rabbit Alexa Fluor Plus 647 (Thermo Fisher Scientific, cat.# A32795): 1:200/1:1000

	donkey anti-mouse Alexa Fluor 488 (Thermo Fisher Scientific, cat.# A21202): 1:200/1:1000<br>donkey anti-goat Alexa Fluor 594 (Thermo Fisher Scientific, cat.# A11058) 1:200
Validation	All antibodies were previously validated by vendors:<br>Oct3/4 mouse anti-mouse (Santa Cruz, cat.# sc-5279): https://www.scbt.com/p/oct-3-4-antibody-c-10?gclid=CjwKCAjwq5-WBhB7EiwAl-HEkuUFTBDEgirOz10qGUglWctHcjRfG2TUH1SdnmLe-GNXckIhDZNKLRoCJbMQAvD_BwE<br>H4K16ac rabbit anti-mouse antibody (Millipore, cat.# 7329): https://www.merckmillipore.com/DK/en/product/Anti-acetyl-Histone-H4-Lys16-Antibody,MM_NF-07-329<br>c-Caspase3 rabbit anti-mouse antibody (Cell Signaling Technology, cat.# 9661S): https://www.cellsignal.com/products/primary-antibodies/cleaved-caspase-3-asp175-antibody/9661<br>FoxO1 rabbit anti-mouse antibody (Cell Signaling Technology, cat.# 2880T): https://www.cellsignal.com/products/primary-antibodies/foxo1-c29h4-rabbit-mab/2880?site-search-type=Products&N=4294956287&Ntt=2880t&fromPage=plp&_requestid=2248978<br>Anti-Lamin B1 antibody - Nuclear Envelope Marker (Abcam, cat#. 16048): https://www.abcam.com/lamin-b1-antibody-nuclear-envelope-marker-ab16048.html<br>Anti-Histone H3 (di methyl K9) antibody (Abcam, cat.# 1220): https://www.abcam.com/histone-h3-di-methyl-k9-antibody-mabcam-1220-chip-grade-ab1220.html<br>Ki67 mouse anti-mouse (BD Pharmingen, cat.# 556003): https://www.bdbiosciences.com/en-be/products/reagents/microscopy-imaging-reagents/immunofluoroscence-reagents/purified-mouse-anti-ki-67.556003<br>pmTOR rabbit anti-human (Abcam, cat.# ab131538): https://www.abcam.com/products/primary-antibodies/mtor-phospho-s2448-antibody-ab131538.html<br>pS6 rabbit anti-mouse antibody (Cell Signaling Technology, cat.# 4858S): https://www.cellsignal.com/products/primary-antibodies/phospho-s6-ribosomal-protein-ser235-236-d57-2-2e-xp-rabbit-mab/4858?_requestid=723559<br>pAKT rabbit anti-mouse antibody (Cell Signaling Technology, cat.# 4060T): https://www.cellsignal.com/products/primary-antibodies/phospho-akt-ser473-d9e-xp-rabbit-mab/4060<br>pAMPK rabbit anti-human antibody (Abcam, cat.# ab23875): https://www.abcam.com/products/primary-antibodies/ampk-alpha-1-phospho-t183--ampk-alpha-2-phospho-t172-antibody-ab23875.html<br>pACC rabbit anti-mouse: (Cell Signaling Technology, cat.# 11818T): https://www.cellsignal.com/products/primary-antibodies/phospho-acetyl-coa-carboxylase-ser79-d7d11-rabbit-mab/11818<br>pULK rabbit anti-mouse antibody: (Cell Signaling Technology, cat.# 14202S): https://www.cellsignal.com/products/primary-antibodies/phospho-ulk1-ser757-d7o6u-rabbit-mab/14202<br>LC3B mouse anti-mouse antibody: (Cell Signaling Technology, cat.# 83506S): https://www.cellsignal.com/products/primary-antibodies/lc3b-e5q2k-mouse-mab/83506<br>LAMP1 rabbit anti-mouse antibody (Cell Signaling Technology, cat.# 99437S): https://www.cellsignal.com/products/primary-antibodies/lamp1-e5n9z-rabbit-mab/99437<br>CPT1A mouse anti-mouse antibody (Abcam, cat.# ab128568): https://www.abcam.com/products/primary-antibodies/cpt1a-antibody-8f6ae9-ab128568.html<br>SOX2 goat anti-mouse antibody (R&D System, cat.# AF2018): https://www.rndsystems.com/products/human-mouse-rat-sox2-antibody_af2018<br>CDX2 mouse anti-mouse antibody (Biogenex, cat.# MU392A-UC): https://biogenex.com/wp-content/uploads/2019/11/932-392M-EN.pdf

Eukaryotic cell lines

Policy information about cell lines and Sex and Gender in Research

Cell line source(s)	ESCs: E14 cells - mouse embryonic stem cells (received from S. Kinkley Lab, MPIMG).<br>TSCs: mouse trophoblast stem cells (received from M. Zernicka-Goetz Lab).<br>MEFs: mouse embryonic fibroblasts (derived in house from CD1 embryos).
Authentication	Cell lines used in this study were sextyped.
Mycoplasma contamination	Cell lines tested frequently negative for mycoplasma contamination.
Commonly misidentified lines<br>(See ICLAC register)	Cell lines used in this paper are not listed in the ICLAC register.

Animals and other research organisms

Policy information about studies involving animals; ARRIVE guidelines recommended for reporting animal research, and Sex and Gender in Research

Laboratory animals	CD1 or b6d2F1 were used. All animal experiments were performed according to local animal welfare laws and approved by the local authority Landesamt für Gesundheit und Soziales (license numbers ZH120, G0284/18, G021/19, and G0243/18).
Wild animals	No wild animals were used in this study.
Reporting on sex	Sex of the embryos has not been considered in the anaylses.
Field-collected samples	No field-collected samples were used in this study.
Ethics oversight	All animal experiments were performed according to local animal welfare laws and approved by local authorities (covered by LaGeSo licenses ZH120, G0284/18, G021/19, and G0243/18).

Note that full information on the approval of the study protocol must also be provided in the manuscript.
